# Novel Mutation of Interferon-γ Receptor 1 Gene Presenting as Early Life Mycobacterial Bronchial Disease

**DOI:** 10.1177/2324709616675463

**Published:** 2016-11-08

**Authors:** Maria J. Gutierrez, Neelu Kalra, Alexandra Horwitz, Gustavo Nino

**Affiliations:** 1 Johns Hopkins University, Baltimore, MD, USA; 2 Allergy Associates of La Crosse, Onalaska, WI, USA; 3 Pennsylvania State University, Hershey, PA, USA; 4 Children’s National Medical Center, Washington, DC, USA; 5 George Washington University, Washington, DC, USA

**Keywords:** primary immunodeficiency, interferon-γ receptor 1, mycobacterial infections, endobronchial disease, innate immunity

## Abstract

Mendelian susceptibility to mycobacterial diseases (MSMD) are a spectrum of inherited disorders characterized by localized or disseminated infections caused by atypical mycobacteria. Interferon-γ receptor 1 (IFNGR1) deficiency was the first identified genetic disorder recognized as MSMD. Mutations in the genes encoding IFNGR1 can be recessive or dominant and cause complete or partial receptor deficiency. We present the case of a 2½-year-old boy with a history of recurrent wheezing, diagnosed with endobronchial mycobacterial infection. Immunological workup revealed a homozygous nonsense mutation in the IFNGR1 gene, a novel mutation predicted in silico to cause complete IFNGR1 deficiency. This case demonstrates that (*a*) Interferon-γ receptor deficiency can present resembling common disorders of the lung; (*b*) mycobacterial infections should be suspected when parenchymal lung disease, hilar lymphadenopathy, and endobronchial disease are present; and (*c*) high index of suspicion for immunodeficiency should be maintained in patients with disseminated nontubercular mycobacterial infection.

## Introduction

Interferon-γ receptor 1 (IFNGR1) deficiency is a rare immune deficiency characterized by selective susceptibility to mycobacterial disease caused by genetic mutations in the IFNGR1 gene.^1^ It comprises about 8% of patients in a group of diseases referred collectively as to Mendelian susceptibility to mycobacterial disease (MSMD) syndromes.^2^ Patients with MSMD feature genetic defects in genes encoding components of the interleukin (IL)-12/23-IFN-γ (interferon-γ) axis, a critical mechanism in the clearance of intracellular infections.^3^

The IFNGR1 gene maps to the chromosome 6q23.3. It is composed of 22 868 base pairs arranged in 7 exons.^4^ On IFN-γ binding, IFNGR1 chain induces the assembly of the IFN-γ receptor (an IFNGR1 and IFNGR2 heterodimer) with subsequent activation of constitutively associated Janus kinases 1 and 2 (Jak1/2) and the downstream signal transducer and activator of transcription 1 (STAT 1) mediated gene transcription.^5^ In the normal host, mycobacteria typically induce IL-12 production by macrophages, which, in turn, trigger IFN-γ production. In patients with IFNGR1 deficiency, macrophages fail to activate on IFN-γ stimulation from T- and NK-cells, rendering the host susceptible to mycobacteria and intramacrophagic microorganisms.^2,5^

Clinically, the characterization of IFNGR1 deficiency–associated mutations translates in important differences in disease severity and treatment approach. Complete autosomal recessive IFNGR1 deficiency is characterized by early onset of disseminated life-threatening infections by low-virulent mycobacteria, lack of response to IFN-γ cytokine replacement therapy, and high mortality.^6,7^ To date hematopoietic stem cell transplant is the only curative therapy available for these patients.^6^ Conversely, the clinical phenotype of the autosomal recessive partial and autosomal dominant forms is milder, usually with later onset, less severe infections, favorable response to IFN-γ and antibiotic therapy, and better survival rates without hematopoietic stem cell transplant.^7,8^

In this article, we describe the case of a toddler boy with a history of recurrent wheezing found to have an invasive endobronchial mycobacterial infection. Genetic testing demonstrated a homozygous c.672 A>G nonsense mutation in exon 5 of the IFNGR1 gene, a novel mutation predicted to cause a stop codon and complete IFNGR1 deficiency. The variant c.672G>A, detected in this patient, is novel and has not been previously described in the literature. This case also illustrates that IFNGR1 deficiency can present with common respiratory symptoms and endobronchial disease in early childhood.

## Case Report

A 2½-year-old boy was admitted with a history of approximately 1 month of recurring fever, productive cough, and intermittent episodes of wheezing and dyspnea. He had been previously treated with azithromycin and cefdinir with only partial improvement. His history was remarkable for recurrent episodes of wheezing and cough approximately every 4 to 6 weeks since the age of 15 months. He had been treated with short courses of oral steroids and antibiotics intermittently with improvement. Otherwise he was a healthy well-developed boy. He was born at full term after an uneventful pregnancy. His family history was unremarkable and there was no history of consanguinity.

At the time of presentation, he was tachypneic and tachycardic with a normal pulsoximetry on exam. His chest exam revealed bilateral wheezing. His abdomen was soft and nondistended with no visceromegaly. He had no clubbing. His physical exam was otherwise unremarkable. Laboratory analysis revealed mild anemia with a hemoglobin of 8.8 g/dL (age-matched control range 11.5-13.5 g/dL). He had a white cell count of 14.73 K/uL with normal absolute neutrophil and lymphocyte counts (Table 1). Basic metabolic panel and liver function tests were within normal limits.

**Table 1.** Summary of Laboratory Findings^a^.

Test	Patient’s Results	Age-Matched Control Range
White cell count	14.73 K/uL	5.5-17 K/uL
Hemoglobin	8.8 g/dL	11.5-13.5 g/dL
Platelets	282 K/uL	172-440 K/uL
Neutrophil count	11.04 K/uL	1.5-8.5 K/uL
Lymphocyte count	2.81 K/uL	2.0-9.5 K/uL
CD3 T cells	61%	56% to 75%
CD3+ CD4+ T cells	31%	28% to 47%
CD3+ CD8+ T cells	20%	16% to 30%
Maximum proliferation of PHA as % CD45	58.9%	≥49.9%
Maximum proliferation of PHA as % CD3	64.3%	≥58.5%

a Patient had mild chronic anemia with otherwise normal T and B blood cell counts and lymphocyte subpopulations. Lymphocyte proliferation to phytohemagluttinin (PHA) yielded normal values. Dihydrorhodamine test for chronic granulomatous disease showed normal oxidative burst.

**Figure 1.**
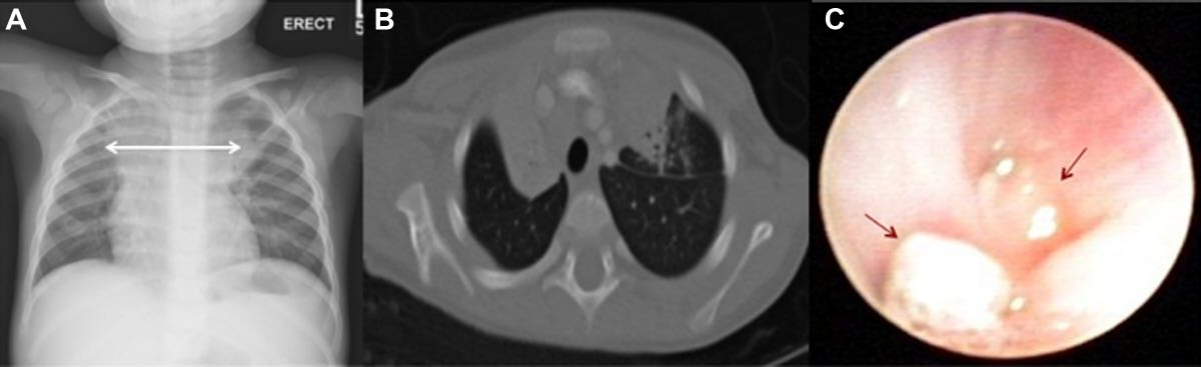
Radiographic and bronchoscopic findings. (A) Mediastinal widening on initial chest radiograph. (B) Extensive mediastinal lymphadenopathy, right main bronchus compression with right upper lobe collapse on chest computed tomography scan during hospital admission. (C) Endobronchial masses found during bronchoscopy. Mycobacterium *avium-intracellulare* complex was isolated from endobronchial granulation tissue.

Chest-X-ray revealed mediastinal widening (Figure 1A). A computed tomography scan of the chest showed extensive mediastinal lymphadenopathy with compression of the right bronchus and collapse of the right upper lobe (Figure 1B). A bronchoscopy revealed cauliflower-like endobronchial masses with one occluding the right main bronchus (Figure 1C). Despite antibiotic therapy, the patient remained febrile and with active respiratory symptoms. Because of a high suspicion of possible mycobacterial infection, empiric therapy was initiated. Mycobacterium *avium-intracellulare* complex was isolated from endobronchial granulation tissue, and bronchoalveolar lavage fluid. After initiation of anti-mycobacterial therapy the patient improved clinically. He was discharged from the hospital on therapy with isoniazid, ethambutol, azithromycin, and rifampicin. He remained afebrile and right upper lobe atelectasis had resolved at follow-up after 4 weeks of therapy.

**Figure 2.**
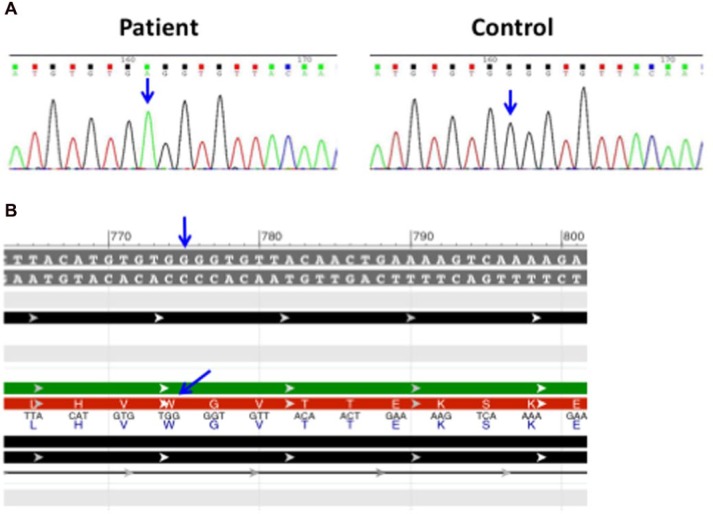
(A) Sequence analysis identified a nonsense homozygous c.672G>A mutation in the IFNGR1 gene. This mutation is predicted to produce a stop codon (TGA) with the resulting protein truncation. (B) Wild type DNA and protein sequences. In the normal protein, the codon TGG encodes for a Tryptophan residue (W) at position 224 (p.Trp224x).

An invasive infection by Mycobacterium *avium-intracellulare* complex raised the concern for an underlying immune defect. Initial immunologic evaluation showed normal T, B, and NK lymphocyte counts with CD4+ CD8+ subpopulations within the normal age-range. Dihydrorhodamine test for chronic granulomatous disease showed normal oxidative burst. Lymphocyte proliferation was preserved in response to mitogen phytohemagluttinin. Genetic analysis demonstrated a nonsense homozygous c.672G>A mutation in the IFNGR1 gene (Figure 2A). We used publicly available software to simulate the potential deleterious effect of the found mutation.^9,10^ Our in silico modeling predicted the c.672G>A substitution to produce a stop codon at the end of exon 5 causing an amino acid change (p.Trp224X) and truncation in the extracellular domain of IFNGR1 likely to produce complete deficiency (Figure 2B). Additional functional studies were unavailable to us as patient transferred to another institution for further care.

## Discussion

We report a case of a 2½-year-old boy presenting with recurrent asthma-like symptoms and a mediastinal mass. Bilateral endobronchial lesions and extensive lymphadenopathy secondary to an atypical mycobacterial infection were found. Subsequently, an underlying primary immunodeficiency associated with a novel nonsense homozygous mutation in the IFNGR1 gene was characterized.

IFNGR1 deficiency can be inherited as an autosomal recessive or an autosomal dominant trait. Functionally, the defect may be partial or complete based on whether the defective receptor is expressed on the cell surface and can bind IFN-γ.^2,5,8^ The autosomal recessive complete deficiency is the rarest but most severe disease form. Approximately 32 individuals carrying 25 mutations have been diagnosed with this variant worldwide.^2^ Most identified mutations to date involve the extracellular domain of the receptor (exons 1 to 5), which usually results in complete absence of protein expression.^1,6,7^ In contrast, mutations associated with autosomal dominant IFNGR1 deficiency have been found in parts of the gene encoding for the intracellular segment of the receptor (last part of exon 6 and exon 7).^1,7^ These mutations produce defective intracellular signaling and receptor recycling but allow the expression of a partially functioning receptor on the cell surface.^5,8^ These differences translate in a milder clinical course, later onset, and response to IFN-γ replacement, which along with antibiotic prophylaxis are the first line of therapy.^6-8^

**Figure 3.**
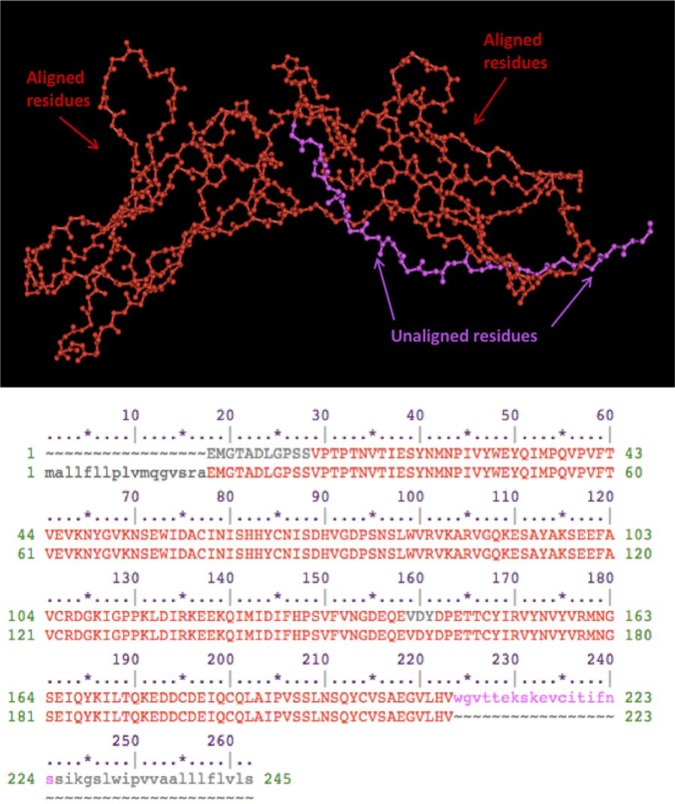
In silico modeling and alignment of the wild type and mutated IFNGR1 extracellular domain. The red segment represents the aligned 223 residues before the protein truncation. The purple segment corresponds to the unaligned residues of the wild protein after the truncation at position 224. (Cn3D viewer—National Center for Biotechnology Information, The National Library of Medicine.)

We describe a novel nonsense mutation in the IFNGR1 gene that has not been previously described in association with a specific disease phenotype. Our in silico model predicted the c.672G>A mutation present in this patient to produce a stop codon at the end of exon 5 with the corresponding amino acid change (p.Trp224X; Figure 2B). This mutation is located in the area of the IFNGR1 gene encoding for the receptor’s extracellular domain, predicted to result in a truncated protein and in likely complete IFNGR1 deficiency (Figure 2B and Figure 3). Of note, in silico analyses, although informative in experimental settings, should not replace appropriate testing for clinical purposes. In this specific case, additional functional studies are needed to confirm the diagnosis of complete IFNGR1 deficiency. IFNGR1 staining by flow cytometry yields information on whether the receptor is expressed on the cell surface.^8^ Additional suitable options to evaluate IFNGR1 function include immunoblotting or flow cytometric evaluation of STAT1 phosphorylation after IFN-α and IFN-γ stimulation.^8^ Establishing the residual protein function (partial vs absent) is essential in determining additional therapeutic choices and long-term outcome.

In summary, this case highlights the need of excluding primary immune defects in patients presenting with low-virulent nontubercular mycobacterial infections. In our case, a plain chest radiograph provided the initial diagnostic clue to an atypical cause, underscoring the importance of baseline chest X-ray evaluation of the wheezing child. This case reminds us that causes of intrinsic and extrinsic airway compression should be kept in the differential diagnosis when evaluating patients with asthma-like symptoms. Although monogenic primary immunodeficiencies are rare, they should be fully explored once the initial clinical suspicion arises. Early diagnosis not only affects patients’ prognosis but also proves definitive for other family members who may need genetic counseling. Finally, given that patients with complete and partial IFNGR1 deficiency require radically different therapy, determining the functional effect of genotyping findings in IFNGR1 deficiency cases is crucial and can provide novel insights into the complex genetic mechanisms implicated in the control of IFN-γ receptor function.
